# The effect of shunt surgery on neuropsychological performance in normal pressure hydrocephalus: a systematic review and meta-analysis

**DOI:** 10.1007/s00415-016-8097-0

**Published:** 2016-03-26

**Authors:** Katie A. Peterson, George Savulich, Dan Jackson, Clare Killikelly, John D. Pickard, Barbara J. Sahakian

**Affiliations:** 1 Department of Psychiatry, Addenbrooke’s Hospital, University of Cambridge, Level 4 Box 189, Cambridge, CB2 0QQ UK; 2 MRC Biostatistics Unit, Institute of Public Health, Robinson Way, Cambridge, UK; 3 Department of Clinical Neurosciences, Addenbrooke’s Hospital, University of Cambridge, Cambridge, UK; 4 MRC/Wellcome Trust Behavioural and Clinical Neuroscience Institute, University of Cambridge, Cambridge, UK

**Keywords:** Normal pressure hydrocephalus, Shunt surgery, Cognition, Neuropsychology, Neuropsychological tests

## Abstract

We conducted a systematic review of the literature and used meta-analytic techniques to evaluate the impact of shunt surgery on neuropsychological performance in patients with normal pressure hydrocephalus (NPH). Twenty-three studies with 1059 patients were identified for review using PubMed, Web of Science, Google scholar and manual searching. Inclusion criteria were prospective, within-subject investigations of cognitive outcome using neuropsychological assessment before and after shunt surgery in patients with NPH. There were statistically significant effects of shunt surgery on cognition (Mini-Mental State Examination; MMSE), learning and memory (Rey Auditory Verbal Learning Test; RAVLT, total and delayed subtests), executive function (backwards digit span, phonemic verbal fluency, trail making test B) and psychomotor speed (trail making test A) all in the direction of improvement following shunt surgery, but with considerable heterogeneity across all measures. A more detailed examination of the data suggested robust evidence for improved MMSE, RAVLT total, RAVLT delayed, phonemic verbal fluency and trail making test A only. Meta-regressions revealed no statistically significant effect of age, sex or follow-up interval on improvement in the MMSE. Our results suggest that shunt surgery is most sensitive for improving global cognition, learning and memory and psychomotor speed in patients with NPH.

**Electronic supplementary material:**

The online version of this article (doi:10.1007/s00415-016-8097-0) contains supplementary material, which is available to authorized users.

## Introduction

Normal pressure hydrocephalus (NPH) is characterized by a build-up of cerebrospinal fluid (CSF) in the brain despite apparently normal CSF pressure at lumbar puncture [1]. Idiopathic NPH (iNPH) typically occurs in later life and without any obvious cause [2, 3]. Symptoms include gait disturbance, urinary incontinence and progressive dementia [1]. Dementia-related symptoms are characterised by deficits in memory, visuospatial abilities, psychomotor speed and executive function [2, 4–10].

The effect of shunt treatment on cognitive performance in patients with NPH is controversial. While CSF drainage is generally considered to relieve problems with gait and incontinence, cognitive impairment is reported to be the least likely symptom to improve [2]. Rates of cognitive improvement range from 0 to 80 % of patients in a given series [2, 11–14]. However, methodological limitations have been identified which could explain the variability observed between studies. These include unclear patient selection criteria, inconsistent follow up intervals and use of subjective measures of improvement [15]. Additionally, due to the lack of standardized clinical guidelines for assessing cognitive function in this patient group, assessment methods often vary between centres with functional grading scales, clinical rating scales, and neuropsychological testing being employed [15]. Studies that have focused on neuropsychological test performance generally show a beneficial effect of shunt surgery on cognitive function. However, again, the pattern of post-operative neuropsychological improvement varies widely between studies [e.g. 2, 5, 9, 11].

Understanding the neuropsychology of NPH may be useful for differential diagnosis as well as interpretation of outcome following treatment [9]. We combined data from the most frequently used neuropsychological tests in an attempt to determine the effect of shunt surgery on neuropsychological performance in patients with NPH. We included studies using neuropsychological tests to assess cognition before and after shunt surgery. We conducted meta-analyses on pre- and post-operative scores for each test. Additionally, we conducted exploratory analyses to investigate effects of moderator variables on cognitive outcome.

## Methods

### Search strategy

A systematic search of the electronic databases PubMed and Web of Science was conducted in October 2015 using the key words: ‘NPH’, ‘normal pressure hydrocephalus’, ‘cognition’, ‘shunt outcome’, ‘neuropsychological outcome’ and ‘neuropsychological assessment’ (separately and in combination) for studies published before October 2015. Due to the limited pool of papers recovered, Google Scholar was included in the search strategy. Reference lists of relevant studies were searched manually. Our review did not have a registered protocol but followed the Preferred Reporting Items for Systematic Reviews and Meta-Analyses (PRISMA) statement [16].

### Study selection

#### Selection of studies

Titles and abstracts of articles were scanned independently by two researchers to identify articles to retrieve in full. Disagreement was dealt with by discussion including a third person.

#### Inclusion criteria

Inclusion criteria were: (1) prospective investigations of cognitive outcome following shunt surgery; (2) patients were adults with a diagnosis of NPH; (3) within-subjects design; and (4) report of pre- and post-operative neuropsychological test scores.

#### Exclusion criteria

Exclusion criteria were: (1) case studies; (2) studies which did not use neuropsychological tests; (3) used neuropsychological tests which were not analysed based on insufficient data; (4) reported composite scores. One study [12] was excluded due to patient overlap with Poca et al. (2004) [17]. Three other papers [18–20] were excluded due to likely patient overlap with other papers that involve larger patient numbers and were included in the review and the analyses that follow.

### Primary outcome measures

Meta-analyses were conducted on pre-operative and “difference” scores in seven neuropsychological tests: the Mini-Mental State Examination (MMSE); the Rey Auditory Verbal Learning Test (RAVLT) total verbal recall and delayed verbal recall subtests; backwards digit span; phonemic verbal fluency; trail making test A (TMT-A); and trail making test B (TMT-B). These were selected as each had at least five studies providing supporting data. Follow-up intervals ranged from 3 to 12 months post-shunt (Table 1). The majority of studies reported follow-up data from one post-operative assessment period. However, one study reported outcome data from more than one post-operative assessment [21]. In this case, data from the earliest follow-up assessment (3 months) were included. Analyses were performed using Stata v13.

**Table 1:** Characteristics of the studies included in meta-analyses

Study	Patient *n*	Patient selection	Age of patients mean (SD) years	Follow-up interval	% males
Andrén et al. [23]	69^c^	Patients with idiopathic NPH	70 (48–84)^a^	3 months	54
Duinkerke et al. [2]	10	Patients with idiopathic NPH who showed improvement in at least one clinical symptom with temporary lumbar drainage	70.9 (10.26)	6–12 months	40
Foss et al. [24]	27	Patients with idiopathic NPH	72 (46–81)^a^	6–9 months	29.6
Gleichgerrcht et al. [5]	10	Patients with idiopathic NPH who showed clinical response to continuous CSF drainage	69.4 (9.3)	6–8 months	70
Hellström et al. [11]	47	Patients with idiopathic NPH	73 (24–84)^a^	3 months	47
Hellström et al. [21]	142	Patients with idiopathic NPH	72.5 (30–87)^a^	3 months	51
Hiraoka et al. [25]	11	Patients with idiopathic NPH	77.9 (4.1)	3 months	40
Iddon et al. [6]	11	Patients with idiopathic NPH	69.64 (6.14)	6 months	72.7
Katzen et al. [7]	12	Patients with idiopathic NPH	74.92 (7.72)	6 months	33.3
Kazui et al. [26]	49^c^	Patients with idiopathic NPH	76.4 (4.4)	3 months	41
Lundin et al. [27]	35	Patients with idiopathic NPH	73 (49–81)^b^	3 months	45.7
Mataró et al. [10]	8	Patients with idiopathic NPH	73.4 (6.8)	6 months	50
Mataró et al. [28]	18	Patients with idiopathic NPH	74.56 (7.06)	6 months	50
Moriya et al. [29]	32	Patients with idiopathic NPH	73.7 (6.8)	12 months	71.9
Peterson et al. [30]	22	Patients with NPH	68.3 (10.8)	3–9 months	63.6
Poca et al. [17]	43	Patients with idiopathic NPH	71.1 (6.9)	6 months	69.8
Saito et al. [8]	32	Patients with idiopathic NPH who showed ≥ 1 point reduction on the total iNPH Grading Scale following shunt surgery	75.7 (4.5)	12 months	50
Savolainen et al. [31]	51	Patients with idiopathic NPH	67.5	3–12 months	52.9
Solana et al. [9]	185	Patients with idiopathic NPH	73.96 (6.3)	6 months	60
Stambrook et al. [32]	14	Patients with NPH	66.0 (14.16)	Mean = 23.73 weeks	64.3
Thomas et al. [14]	42	Patients with idiopathic NPH	73 (10)	3–9 months	45.2
Virhammar et al. [33]	173	Patients with idiopathic NPH	74 (54–88)^a^	12 months	53
Yamamoto et al. [34]	16	Patients with idiopathic NPH	75.8 (4.9)	3 months	50

*CSF* cerebrospinal fluid, *NPH* normal pressure hydrocephalus

^a^Median (range)

^b^Mean (range)

^c^Treatment-as-normal group

### Statistical analysis

Random-effects meta-analyses were performed using the average difference between pre-operative and post-operative scores (difference scores) as outcome data and the standard method of DerSimonian and Laird [22]. Average difference scores were provided by some studies, while for others these were calculated from average pre-operative and post-operative scores. In all meta-analyses, a positive difference indicates that the average post-operative score is more than the pre-operative score. Hence in some meta-analyses positive estimates indicate patient improvement and in others positive estimates indicate deterioration. However, pooled estimates from all seven meta-analyses lie in the direction where post-operative scores are better than the corresponding pre-operative score.

To include all studies providing relevant outcome data, medians were used as means where these were reported. Where interquartile ranges or ranges were reported instead of standard deviations, these were converted to standard deviations by assuming that their bounds correspond to appropriate quantiles from a normal distribution.

The within-study variances of the average differences were calculated using the reported standard deviations and the numbers of patients. For studies that did not give average difference scores directly, we calculated variances of the average pre-operative and post-operative scores in the same way and allowed for a correlation between these two scores when calculating within-study variances of their difference; this is important because scores from the same patients will generally be positively correlated. We assumed a moderate correlation of 0.6 between the average pre-operative and post-operative scores. Our conclusions were robust when assuming alternative correlations of 0.4 and 0.8 (results not shown).

Due to the small numbers of patients comprising the studies, the approximations that underlie the random-effects model are not especially precise. This is evident when, for example, studies’ statements about the statistical significance of their difference scores are not necessarily reflected in the forest plots. Therefore, we carefully assess whether the results are robust below.

Random-effects meta-analyses were also performed using average pre-operative scores to investigate whether instances of lack of improvement were due to ceiling effects. Finally, three random-effects meta-regression models were fitted using the average difference in MMSE as outcome data to assess the evidence that three covariates may be useful predictors of cognitive change.

We did not use any statistical method to assess publication bias. Whilst recognising this as an important issue for meta-analyses, not all studies contribute outcome data to all meta-analyses. Hence the sample sizes are inadequate to assess this issue formally. Furthermore, it is plausible to assume an absence of publication bias in our systematic review. This is because publication bias is usually thought to occur because studies indicating a treatment effect are more likely to be published but our studies do not compare treatment groups in this way.

## Results

### Search results

**Fig. 1:**
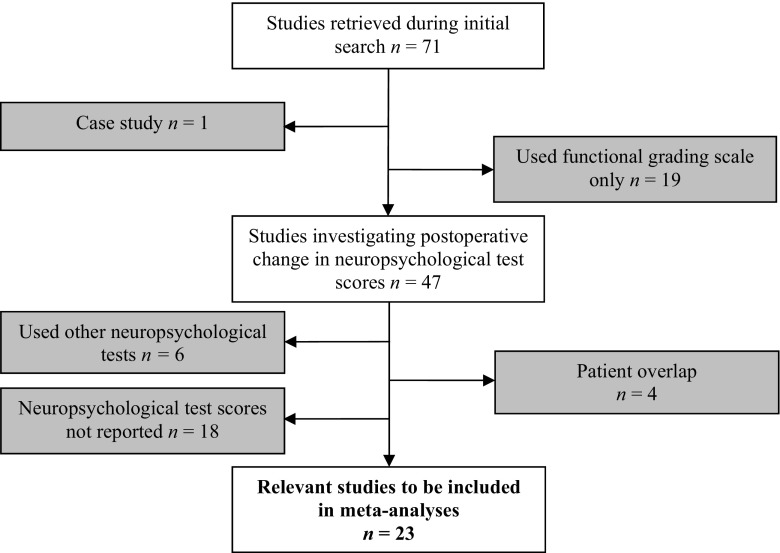
PRISMA flow chart for review

Seventy-one studies were identified following a systematic literature search. Forty-eight were excluded (Fig. 1) and twenty-three met criteria for inclusion in meta-analyses (Table 1). A subset of these studies provide outcome data for each neuropsychological test. Nineteen studies provide outcome data for the MMSE; seven studies provide outcome data for RAVLT total and delayed recall subtests; six studies provide outcome data for backwards digit span; eight studies provide outcome data for phonemic verbal fluency; 13 studies provide outcome data for TMT-A; and nine studies provide outcome data for TMT-B (Table 2; supplementary figures).

**Table 2:** Meta-analyses results

Cognitive domain	Test	*n* of studies	Estimated average pre-shunt score	95 % CI	Estimated average difference	95 % CI	*p*	Cochran’s *Q* (*df*; *p*)	*I* ^2^ (%)	Estimated between-study variance
Global function	MMSE	19	23.10 points	22.13, 24.08	2.20 points	1.45, 2.95	<0.001	99.62 (18; <0.001)	81.9	1.99
Learning and memory	RAVLT total	7	22.73 words	19.86, 25.61	5.64 words	3.86, 7.43	<0.001	14.02 (6; 0.03)	57.2	2.68
	RAVLT delayed	7	1.90 words	1.22, 2.57	1.43 words	0.55, 2.31	0.001	56.33(6; <0.001)	89.3	1.11
Executive function	Backwards digit span	6	2.92 digits	2.38, 3.46	0.36 digits	0.04, 0.67	0.03	38.61 (5; <0.001)	87.0	0.12
	Phonemic verbal fluency	8	19.67 words	13.60, 25.74	2.73 words	0.84, 4.63	0.005	10.55 (7; 0.16)	33.6	2.32
	TMT-B	9	293.03 s	221.09, 364.97	−43.46 s	−83.23, −3.70	0.03	35.89 (8; <0.001)	77.7	2494.80
Psychomotor speed	TMT-A	13	132.48 s	108.48, 156.49	−25.90 s	−36.11, −15.69	<0.001	18.78 (12; 0.09)	36.1	104.03

*MMSE* Mini-Mental State Examination, *RAVLT* Rey Auditory Verbal Learning Test, *TMT* trail making test

### Average pre-operative scores

The estimated average pre-operative score for each test was as follows: MMSE = 23.10 points (95 % CI 22.13–24.08); RAVLT total verbal recall = 22.73 words (95 % CI 19.86–25.61); RAVLT delayed verbal recall = 1.90 words (95 % CI 1.22–2.57); backwards digit span = 2.92 digits (95 % CI 2.38–3.46); phonemic verbal fluency = 19.67 words (95 % CI 13.60–25.74); TMT-B = 293.03 s (95 % CI 221.09–364.97); and TMT-A = 132.48 s (95 % CI 108.48–156.49) (Table 2).

### Average difference scores (pre- to post-operative)

There was a statistically significant effect of shunt surgery on cognition (MMSE: pooled average difference = 2.20 points, 95 % CI 1.45–2.95, *p* < 0.001; *I*^2^ = 81.9 %, Supplemental Figure 1), memory (RAVLT total verbal recall: pooled average difference = 5.64 words, 95 % CI 3.86–7.43; *p* < 0.001, *I*^2^ = 57.2 %, Supplemental Figure 2; delayed verbal recall: pooled average difference = 1.43 words, 0.55–2.31; *p* = 0.001, *I*^2^ = 89.3 %, Supplemental Figure 3), executive function (backwards digit span: pooled average difference = 0.36 digits, 0.04–0.67; *p* = 0.03, *I*^2^ = 87.0 %, Supplemental Figure 4; phonemic verbal fluency: pooled average difference = 2.73 words, 95 % CI 0.84–4.63, *p* = 0.005, *I*^2^ = 33.6 %, Supplemental Figure 5; TMT-B: pooled average difference = −43.46 s, 95 % CI −83.23 to −3.70, *p* = 0.03, *I*^2^ = 77.7 %, Supplemental Figure 6), and psychomotor speed (TMT-A: pooled average difference = −25.90 s, 95 % CI −36.11 to −15.69; *p* < 0.001, *I*^2^ = 36.1 %; Supplemental Figure 7).

### Interpretation of difference scores

All analyses show statistically significant estimated average differences in the direction of improvement following shunt surgery in the presence of moderate to high heterogeneity (Table 2). There is strong evidence for five of these average differences: MMSE (*p* < 0.001), RAVLT total verbal recall (*p* < 0.001), RAVLT delayed verbal recall (*p* = 0.001), phonemic verbal fluency (*p* = 0.005) and TMT-A (*p* < 0.001). The remaining tests (backwards digit span, and TMT-B) show weaker significance levels (*p* = 0.03; 0.03; respectively). Given the problems associated with repeated testing, and because of the approximations made by the statistical methods used, we suggest that the statistical significance of these two tests be treated with caution and we do not view them as robust. The I^2^ statistics range from 33 to 90 %, indicating considerable between-study heterogeneity in all outcomes and meaning that the studies estimate substantially different effects. This means that any single study is susceptible to producing results that differ from the estimated average differences. The pooled estimates must therefore be interpreted as population average differences, and not study specific differences, in accordance with the random effects model for meta-analysis.

Visual analysis of the forest plots supports the above interpretations. For all forest plots, average scores across studies are in very good directional agreement with the estimated average difference scores, but this is less clear for backwards digit span and TMT-B.

### Moderator variables

All nineteen studies included in the analysis of moderator variables provided information about average age, time-to-retest and % males. Random effects meta-regressions using average difference in MMSE as outcome data were all non-significant (Table 3). We did not find evidence that average age, time-to-retest or sex predict improvement in the MMSE.

**Table 3:** Meta-regressions of average difference of MMSE on moderator variables

Covariate	Estimate	Standard error	*p* value	95 % CI
Time-to-retest (months)	0.01	0.13	0.96	−0.24, 0.25
Av. age (years)	−0.15	0.15	0.29	−0.44, 0.13
% male	0.05	0.03	0.09	−0.01, 0.11

## Discussion

The aim of the current review was to determine the effect of shunt surgery on neuropsychological test performance in patients with NPH. Twenty-three studies were eligible for inclusion within one or more meta-analyses. Meta-analyses were conducted on average pre-operative and average “difference” scores for seven neuropsychological tests. Statistically significant estimated average difference scores were observed for all tests in the direction of improvement following shunt surgery. However, detailed examination of the results suggested robust evidence for improved MMSE, RAVLT total verbal recall, RAVLT delayed verbal recall, phonemic verbal fluency and TMT-A only. Meta-regressions revealed no significant effects of age, time-to-retest or sex on average MMSE difference score.

### Memory

Post-shunt improvement in memory is frequently reported in patients with NPH. Significant improvement has been found for visual recall [35, 36], spatial memory [6], and in various subtests of the Wechsler Memory Scale [2, 9, 14, 37]. However, the RAVLT appears to be highly sensitive to cognitive improvement in NPH. We found robust evidence for improvement in the total and delayed verbal recall subtests and significant improvement has also been documented in RAVLT retention score [2, 37].

### Executive function

It is unclear whether executive function improves following shunt surgery. Some studies report significant improvement in the backwards digit span test [5, 9, 11, 28], whilst others report no change [10, 12, 17, 19]. Similarly, improvements in the Stroop test have been observed in some studies [2, 11, 21], but not in others [10, 14, 28, 31]. A ceiling effect has been suggested to underlie the absence of improved executive function [37]. However, studies have found performance in tests of executive function to be disproportionately impaired in NPH patients at baseline [6, 8], and suggested that lack of improvement reflects an irreversible frontal executive impairment.

Only one of three tests of executive function in our meta-analyses showed robust evidence for improvement (phonemic verbal fluency). The remaining two (backwards digit span and TMT-B) had weaker significance levels, and supporting studies did not indicate agreement in the direction of improvement. We performed meta-analyses using average pre-operative scores to investigate whether instances of lack of improvement were due to ceiling effects. The estimated average pre-operative score for backwards digit span was 2.92 digits. Median score in this test by 159 healthy controls in a study by Hellstrom et al. [11] was 4 digits. Estimated average pre-operative score for TMT-B was 293.03 s. Normative data provided by Tombaugh [38] suggests individuals aged 70–74 complete this test in 109.95 s (less time indicates better performance). Estimated average pre-operative scores for both tests indicated that, on average, patients were impaired in these tests compared to age-matched normative data. This suggests that ceiling effects cannot explain the lack of robust evidence for improvement in these tests following shunt. Nevertheless, robust evidence for improvement was observed for phonemic verbal fluency. However, phonemic verbal fluency is simplistic compared to executive tests with strategic or problem solving aspects. Therefore, improvement in this test likely reflects improved attentional capacity rather than higher level executive function. Overall, given the tests we could include in the analyses, our results do not provide strong evidence for improvement in executive function following shunt surgery, tentatively supporting the hypothesis that executive impairment in NPH may reflect irreversible damage to fronto-subcortical connectivity. However, further investigation using more sensitive tests of executive function are needed as improvements in this domain have been found [5].

### Psychomotor speed

We found good evidence for improvement in psychomotor speed, as measured by the TMT-A. Due to lack of data, we were unable to include other tests of psychomotor speed, although improvements have also been documented in the Grooved pegboard test [21], the Purdue pegboard test [10], and the Line-tracing test [14].

### Global cognitive functioning

We found robust evidence for improved performance on the MMSE. This test is commonly used to assess cognitive function in NPH, although results vary with some studies finding significant improvements [14, 28, 34], and others finding no change [5, 8, 12, 17]. A ceiling effect may explain why some studies find no change on the MMSE. High functioning patients can perform well on this test while specific cognitive deficits may be missed unless detailed neuropsychological testing is conducted [39]. Indeed, in their study, Iddon et al. [6] split patients according to their pre-operative MMSE scores. Patients who scored in the dementing range of the MMSE at baseline (<24 points) improved to the normal range post-operatively. However, no significant difference was observed between baseline and outcome scores for patients who did not score in the dementing range at baseline. Therefore, it is important that cognitive assessments include a battery of neuropsychological tests in addition to the MMSE.

### Practice effects

Studies with test–retest control groups provide evidence that improvements following shunt surgery are due to treatment effects rather than practice effects. Katzen et al. [7] found greater improvement in measures of mental tracking speed and sustained attention in shunted iNPH patients than in healthy controls who had undergone repeated testing. Saito et al. [8] found evidence for improvements in executive function following shunt which were not ascribable to practice effects. Furthermore, Solana et al. [40] investigated the effect of testing–retesting in patients with NPH using a battery of neuropsychological tests administered over four consecutive days. No learning effect was observed for any of the tests and it was concluded that improvements following shunt reflect a true treatment effect.

## Predicting improvement

Since shunt surgery is an invasive procedure and patients are often elderly, it is important to identify factors which predict positive outcome following treatment. We found no significant effects of age, sex, or time between shunt and reassessment on outcome in the MMSE. However, this was an exploratory analysis and effects may be observed using other measures of cognitive or functional outcome.

## Extent and duration of improvement

Although cognitive improvement has been observed in patients with NPH following shunt surgery, patients remain impaired in neuropsychological tests compared to age-matched controls. Shunted patients have shown to perform significantly poorer than healthy controls in tests of psychomotor speed, memory and executive function at both three and 12 months post-shunt [11, 21]. We investigated outcome between three and 12 months post-shunt, however, from the available data, we were unable to assess outcome at longer durations. To determine the extent of cognitive recovery, longer-term monitoring of patients is required using multiple post-operative assessments as improvements have been documented as late 5 years post-shunt [41].

## Limitations and methodological considerations

We have not attempted to formally assess the risk of bias because of the difficult nature of determining what constitutes study quality in this area and so leave it to the reader to assess study quality if they wish to consider this issue.

Methodological differences across studies complicate interpretation of results. Variability within tests used meant that our analyses were limited to seven neuropsychological tests when others may show improvement following shunt surgery. Furthermore, higher level executive functions could not be assessed with the restricted set of tests used to date. Additionally, time between shunt and reassessment varied with 3, 6 and 12 month delays being used. Consistency here is pertinent as different patterns of improvement may be seen at different intervals. Improvement may be observed more readily at shorter intervals due to immediate effect of the shunt, whereas initial improvement may be missed at longer intervals due to effects of comorbid disorders or increasing age [9].

## Conclusions

We found evidence for improved performance in global cognitive function, verbal learning and memory and psychomotor speed following shunt surgery. However, we did not find strong evidence for improvement in tests of executive function based on the available data. To clarify these findings, we suggest that there is a need to assess high-level executive functions in patients with NPH before and after shunt surgery. Additionally, longer-term monitoring of patients is required to determine the extent to which cognitive functions may improve. The MMSE, the RAVLT, phonemic verbal fluency and trail making test A may be useful for assessment of cognitive outcome following treatment for NPH.

## Electronic supplementary material

Below is the link to the electronic supplementary material.

Supplementary material 1 (PDF 207 kb)
