# Supplementary material for: The effect of shunt surgery on neuropsychological performance in normal pressure hydrocephalus: a systematic review and meta-analysis
Source: J Neurol. 2016 Mar 26;263:1669–77. doi: 10.1007/s00415-016-8097-0 (PMC4971036; doi:10.1007/s00415-016-8097-0)
Supplement: Supplementary file 1 — Supplementary material 1 (PDF 207 kb) [file 415_2016_8097_MOESM1_ESM.pdf]

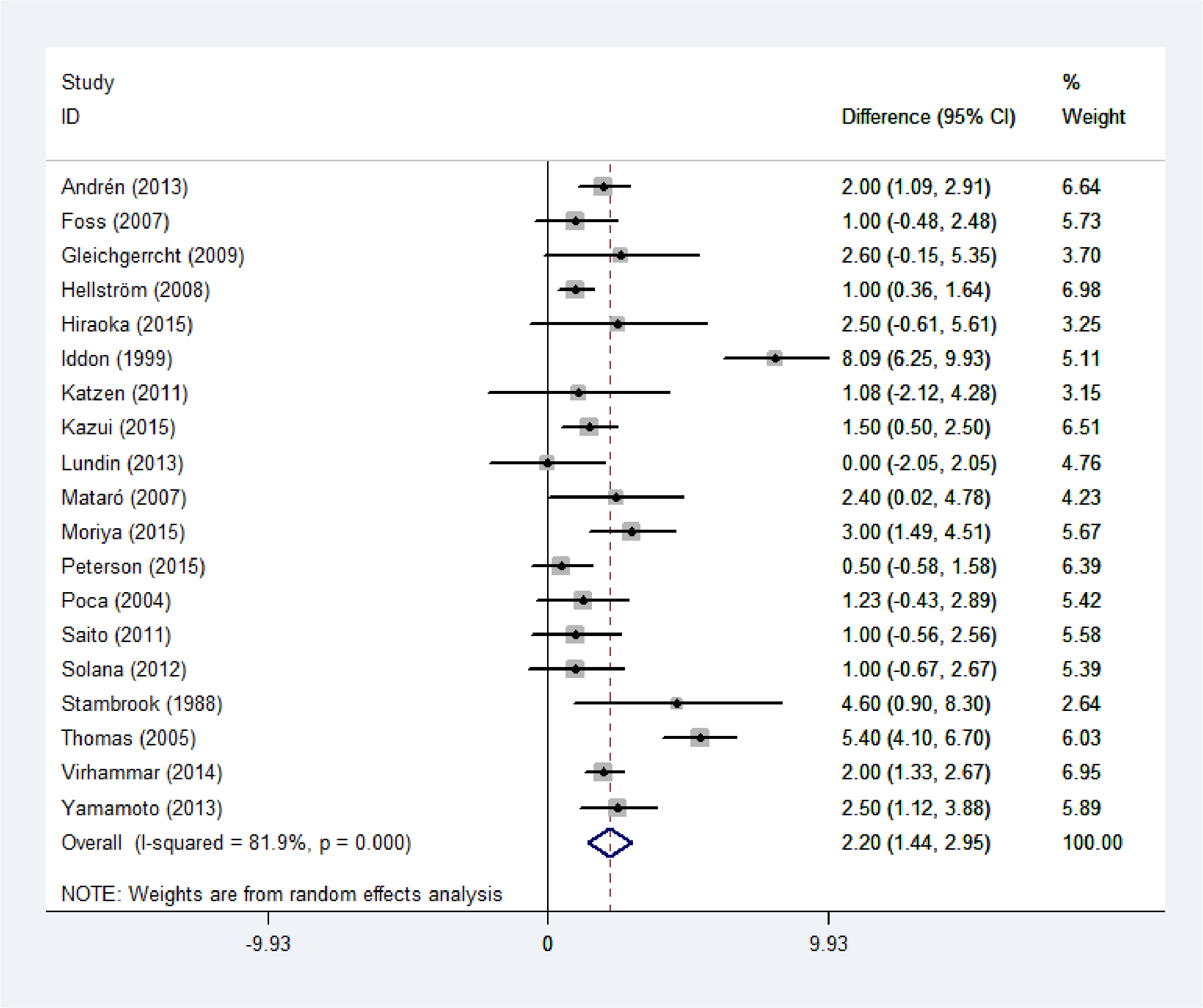

Figure 1. *Forest plot for difference in MMSE. The pooled outcome was determined using a random-effects model. Average differences are shown with 95 per cent confidence intervals.*

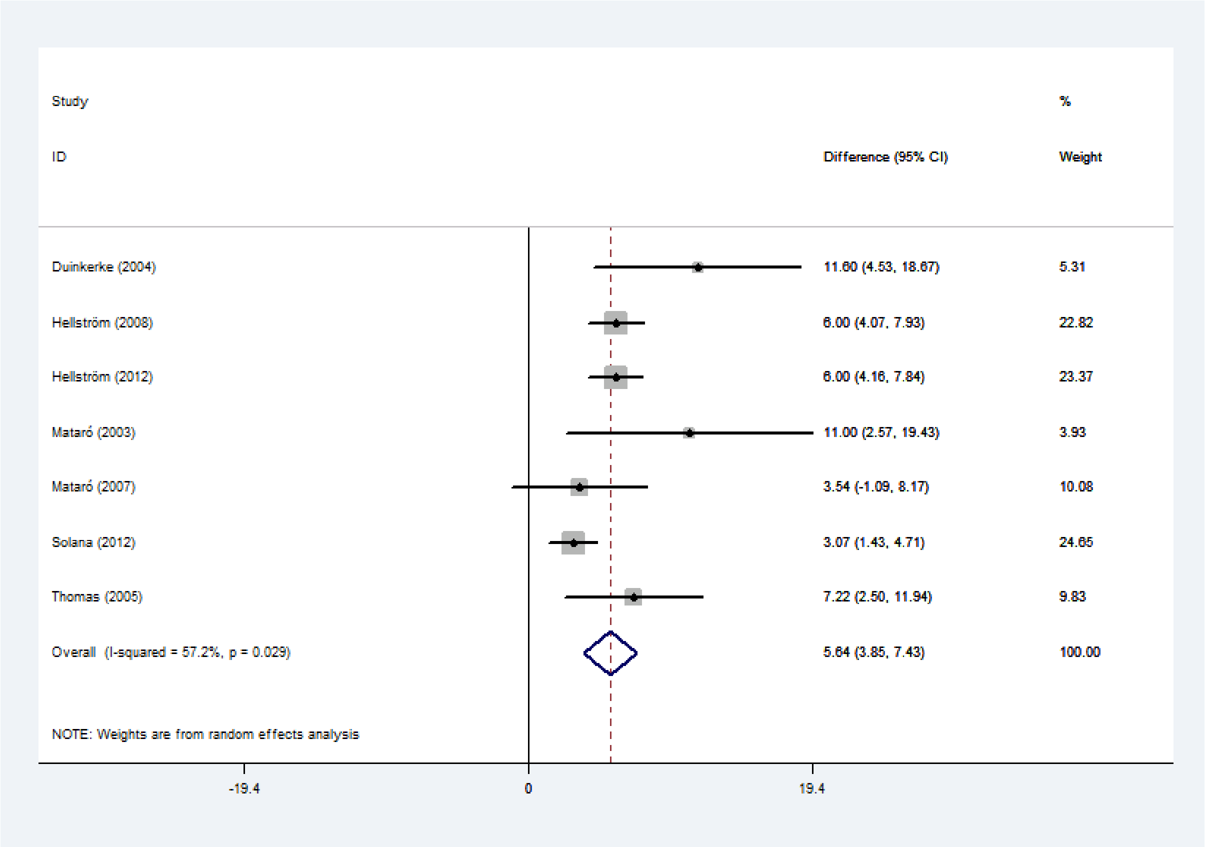

Figure 2. *Forest plot for difference in RAVLT total verbal recall. The pooled outcome was determined using a random-effects model. Average differences are shown with 95 per cent confidence intervals.*

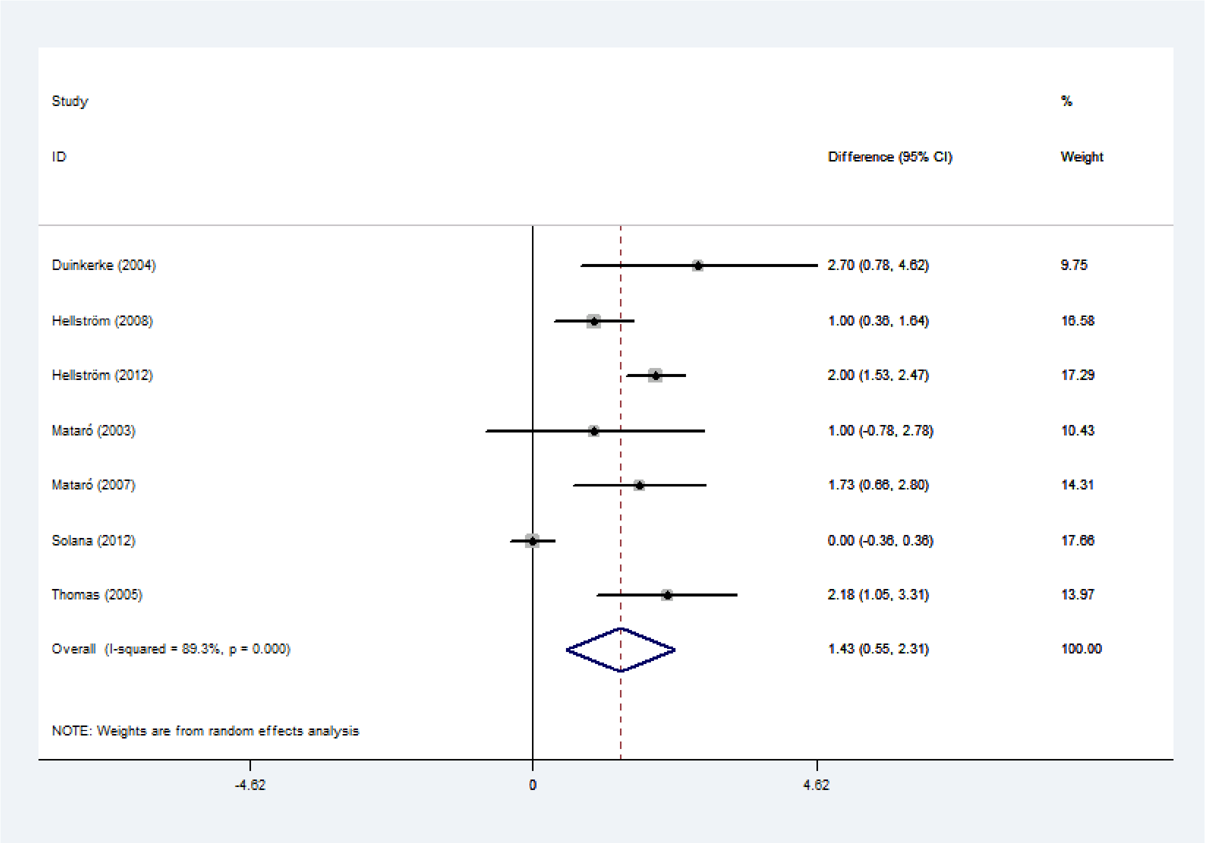

Figure 3. *Forest plot for difference in RAVLT delayed verbal recall. The pooled outcome was determined using a random-effects model. Average differences are shown with 95 per cent confidence intervals.*

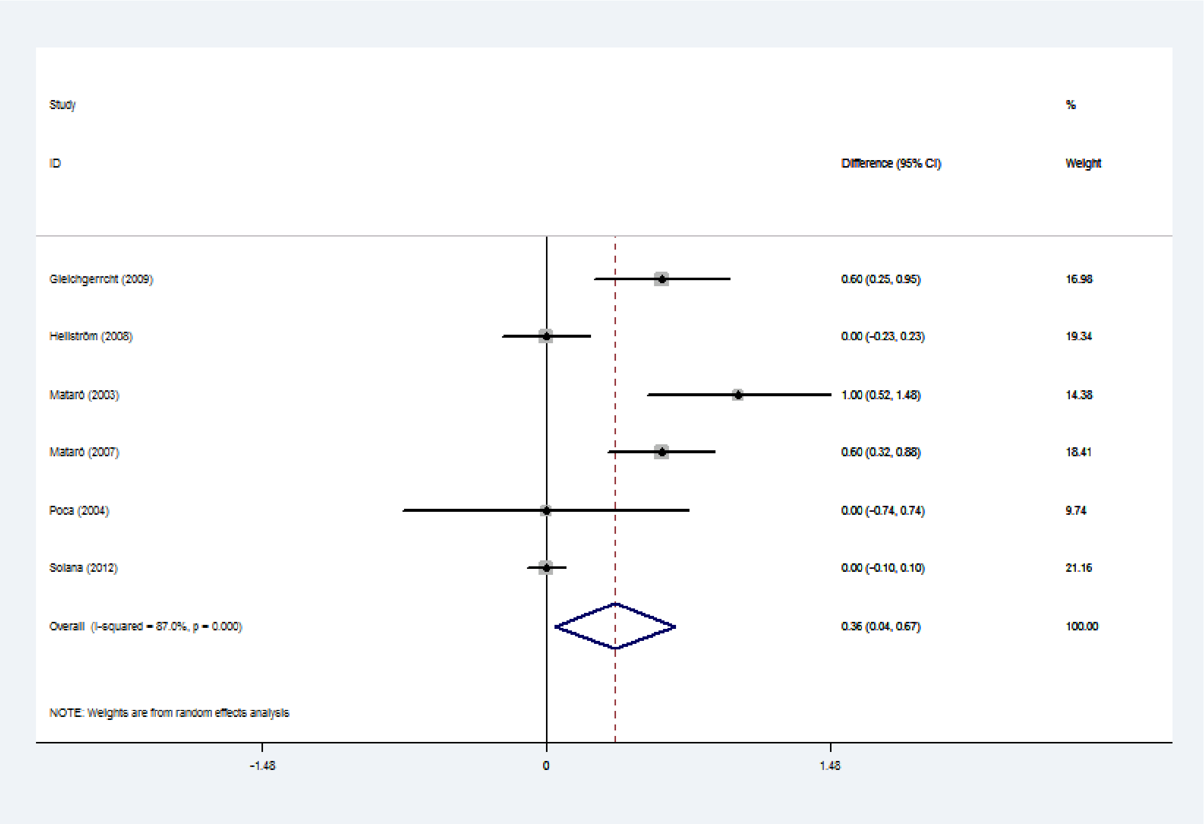

Figure 4. *Forest plot for difference in backwards digit span score. The pooled outcome was determined using a random-effects model. Average differences are shown with 95 per cent confidence intervals.*

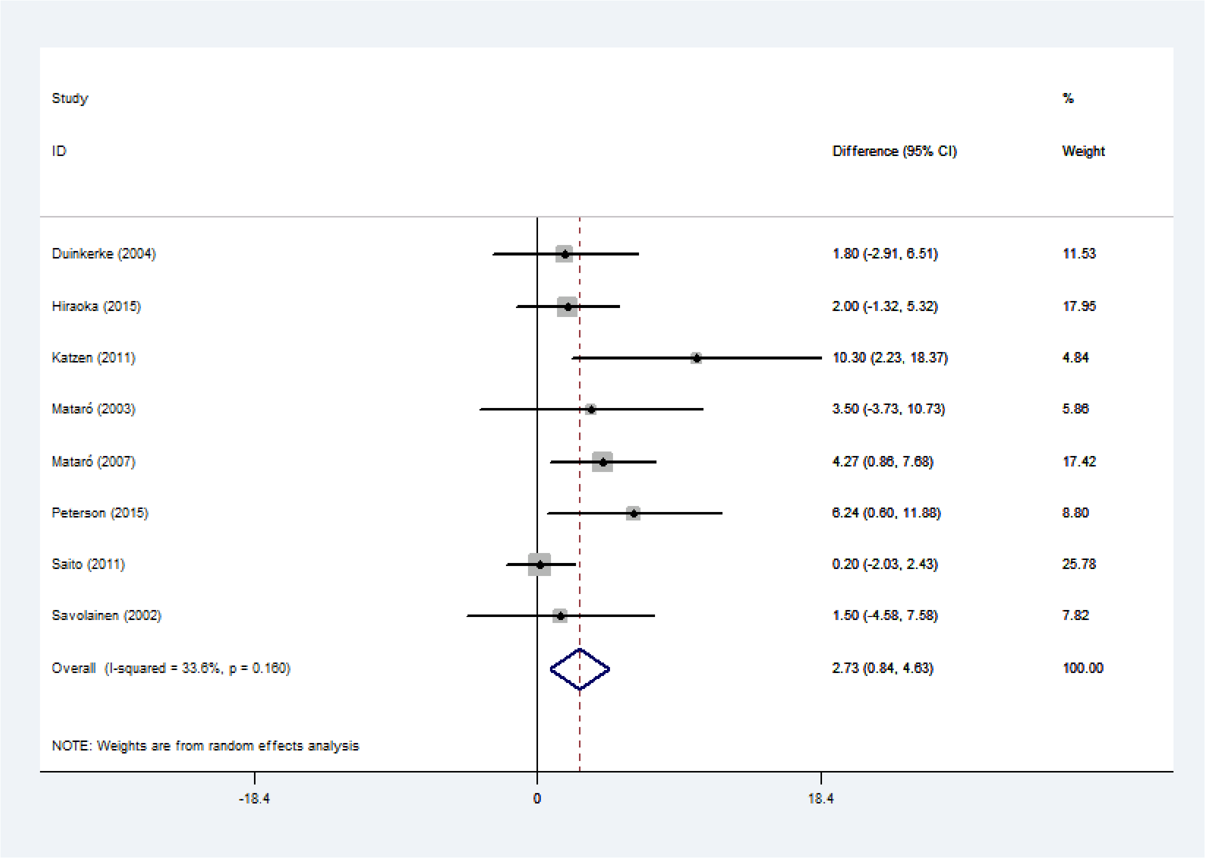

Figure 5. *Forest plot for difference in phonemic verbal fluency. The pooled outcome was determined using a random-effects model. Average differences are shown with 95 per cent confidence intervals.*

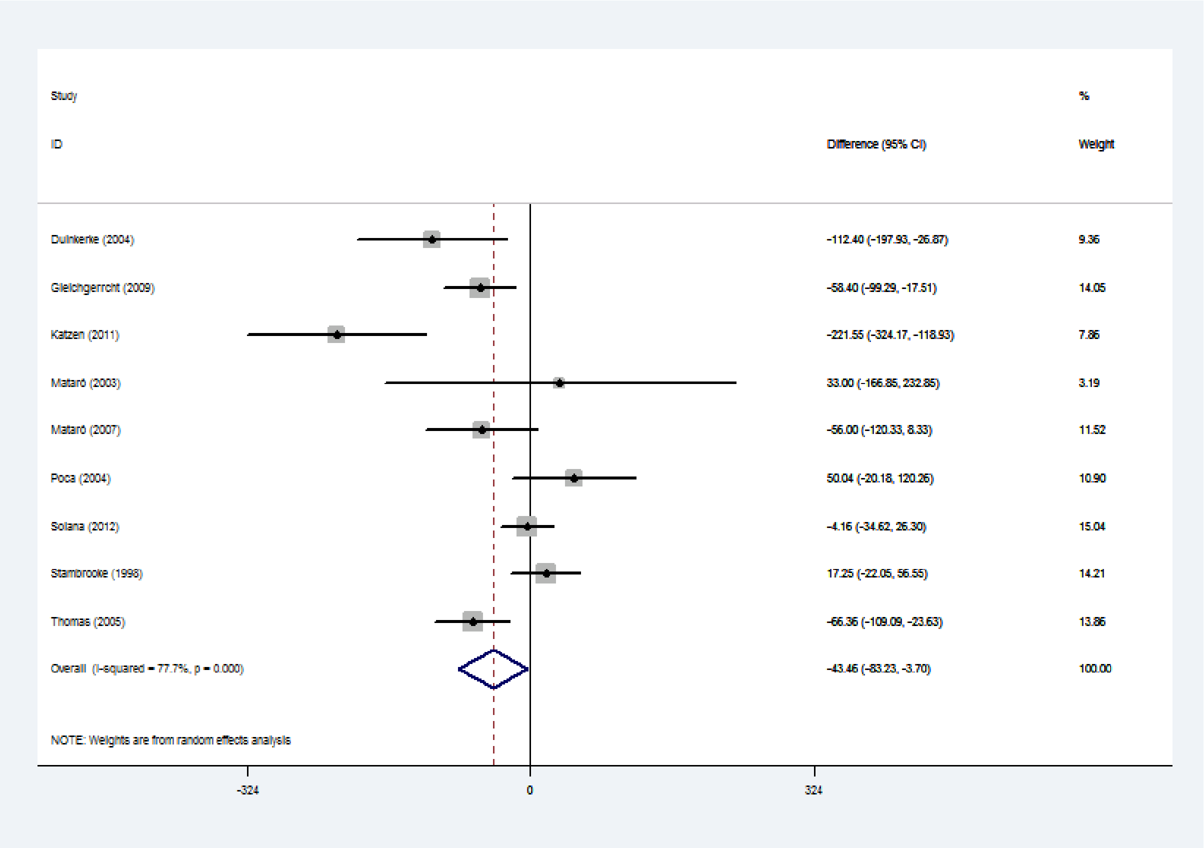

Figure 6. *Forest plot for difference in trail making test B score (seconds to complete). The pooled outcome was determined using a random-effects model. Average differences are shown with 95 per cent confidence intervals.*

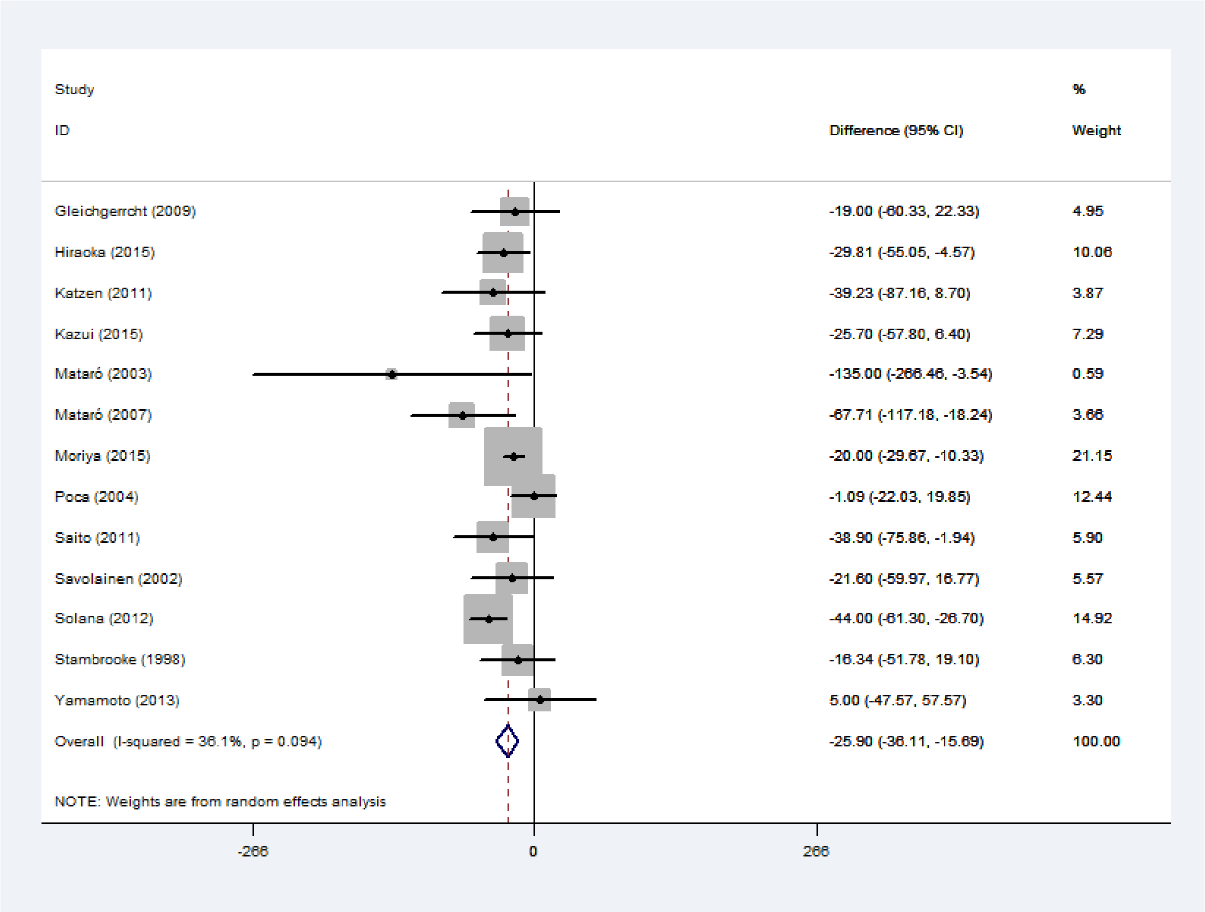

Figure 7. *Forest plot for difference in trail making test A score (seconds to complete). The pooled outcome was determined using a random-effects model. Average differences are shown with 95 per cent confidence intervals.*
